# Synovial CD4+ T-cell-derived GM-CSF supports the differentiation of an inflammatory dendritic cell population in rheumatoid arthritis

**DOI:** 10.1136/annrheumdis-2014-206578

**Published:** 2015-04-28

**Authors:** G Reynolds, J R Gibbon, A G Pratt, M J Wood, D Coady, G Raftery, A R Lorenzi, A Gray, A Filer, C D Buckley, M A Haniffa, J D Isaacs, C M U Hilkens

**Affiliations:** 1 Arthritis Research UK Rheumatoid Arthritis Pathogenesis Centre of Excellence; 2 Musculoskeletal Research Group, Newcastle University, Newcastle-Upon-Tyne, UK; 3 Sunderland Royal Hospital, Sunderland, UK; 4 Department of Rheumatology, Freeman Hospital, Newcastle-Upon-Tyne, UK; 5 Rheumatology Research Group, MRC Centre for Immune Regulation, School of Immunity and Infection, College of Medical and Dental Sciences, University of Birmingham, Birmingham, UK; 6 Haematological Sciences, Institute of Cellular Medicine, Newcastle University, Newcastle-Upon-Tyne, UK

**Keywords:** Rheumatoid Arthritis, T Cells, Inflammation, Cytokines, Autoimmunity

## Abstract

**Objective:**

A population of synovial inflammatory dendritic cells (infDCs) has recently been identified in rheumatoid arthritis (RA) and is thought to be monocyte-derived. Here, we investigated the role and source of granulocyte macrophage-colony-stimulating factor (GM-CSF) in the differentiation of synovial infDC in RA.

**Methods:**

Production of GM-CSF by peripheral blood (PB) and synovial fluid (SF) CD4+ T cells was assessed by ELISA and flow cytometry. In vitro CD4+ T-cell polarisation experiments were performed with T-cell activating CD2/CD3/CD28-coated beads in the absence or presence of pro-Th1 or pro-Th17 cytokines. CD1c+ DC and CD16+ macrophage subsets were flow-sorted and analysed morphologically and functionally (T-cell stimulatory/polarising capacity).

**Results:**

RA-SF CD4+ T cells produced abundant GM-CSF upon stimulation and significantly more than RA-SF mononuclear cells depleted of CD4+ T cells. GM-CSF-producing T cells were significantly increased in RA-SF compared with non-RA inflammatory arthritis SF, active RA PB and healthy donor PB. GM-CSF-producing CD4+ T cells were expanded by Th1-promoting but not Th17-promoting conditions. Following coculture with RA-SF CD4+ T cells, but not healthy donor PB CD4+ T cells, a subpopulation of monocytes differentiated into CD1c+ infDC; a process dependent on GM-CSF. These infDC displayed potent alloproliferative capacity and enhanced GM-CSF, interleukin-17 and interferon-γ production by CD4+ T cells. InfDC with an identical phenotype to in vitro generated cells were significantly enriched in RA-SF compared with non-RA-SF/tissue/PB.

**Conclusions:**

We demonstrate a therapeutically tractable feedback loop of GM-CSF secreted by RA synovial CD4+ T cells promoting the differentiation of infDC with potent capacity to induce GM-CSF-producing CD4+ T cells.

## Introduction

Rheumatoid arthritis (RA) is a chronic disease characterised primarily by synovial inflammation. The presence of autoreactive T cells and antibodies recognising citrullinated self-peptides in the peripheral blood (PB) of patients with RA supports the notion that the disease is initiated by an antigen-specific T-cell response.^1–5^ As efficient activators of antigen-specific T-cell responses, dendritic cells (DCs) have been implicated in disease pathogenesis.^6^^–^10

In humans, two populations of steady-state myeloid DCs exist in blood and tissues subdivided according to differential expression of CD141 and CD1c.^11^ ^12^ Analogous populations exist in mice identified by the markers CD103 and CD11b.^12^ These derive from sequentially restricted precursors in the bone marrow that form a distinct branch from the monocyte lineage.^13–15^ A third population of monocyte-derived inflammatory DCs (infDCs) has been shown to develop in response to a range of insults including leishmaniasis,^16^ influenza,^17^ trypanosomiasis,^18^ listeriosis^19^ and pulmonary aspergillosis.^20^ A human infDC equivalent was recently identified in RA synovial fluid (SF)^21^ that was indistinguishable from steady-state CD1c+ DCs by surface phenotype but with a transcription profile closer to that of monocyte-derived DCs (moDCs). In contrast to inflammatory macrophages, this population efficiently promoted Th17 responses through production of interleukin (IL)-23.

The differentiation requirements for infDCs have not been established but there are two lines of evidence to suggest a critical role for granulocyte-macrophage colony-stimulating factor (GM-CSF). First, GM-CSF is an essential growth factor for in vitro and in vivo DC development.^22^ Second, GM-CSF is found at high concentrations at sites of inflammation in several diseases including asthma,^23^ multiple sclerosis^24^ and RA.^25^ Therapeutic neutralising antibodies targeting GM-CSF have shown promise in phase II clinical trials in RA.^26^

CD4+ T cells are a recognised source of GM-CSF. In experimental autoimmune encephalomyelitis (EAE, a murine model of multiple sclerosis) the production of GM-CSF by CD4+ T cells is necessary and sufficient to render them encephalitogenic.^27^ ^28^ GM-CSF is thought to exert its pathogenic effect in this model by enhancing IL-23 production by CNS-infiltrating CD11c+ DCs and thereby stabilising the Th17 population.^27^ In RA synovial tissue CD4+ T cells colocalise with CD1c+ DCs^29^ suggesting a potential symbiotic interaction which promotes inflammation.

The contribution of CD4+ T cell-derived GM-CSF to murine EAE has created interest in the factors that modulate its production. While polarised Th1, Th2 and Th17 CD4+ T cells can all produce GM-CSF, it has been designated predominantly a Th17 cytokine in mice^28^ as (1) its production is inhibited by the Th1-associated cytokines interferon (IFN)-γ and IL-12; (2) its production is enhanced in Th17 cells by IL-1β and IL-23^27^ ^28^ and (3) it is under the control of the Th17 master transcription factor RORγt^28^ (although this is disputed^27^). The factors that control GM-CSF production by CD4+ T cells in humans are not fully established.

In this study, we investigated the cellular source of GM-CSF in RA-SF and the contribution of RA-SF T-cell-derived GM-CSF in infDC differentiation from PB monocytes. Our data demonstrate that CD4+ T cells are a major source of GM-CSF in RA and that cytokines known to be present in the RA synovium prime them to produce it. Furthermore, we demonstrate that by producing GM-CSF, CD4+ T cells are capable of inducing an infDC phenotype in monocytes.

## Materials and methods

### Patient samples

SF was obtained during therapeutic aspirations. Osteoarthritis (OA) and healthy synovial tissue were obtained from orthopaedic procedures. RA synovial tissue was obtained by ultrasound-guided biopsy. Healthy donor blood was obtained from volunteers. Patients with active RA (defined as a disease activity score (DAS28) >5.1) were recruited from a biological initiation clinic. OA and active RA synovial fibroblasts were cultured from synovial tissue obtained by arthroscopy and used between passage 4–8.^30^ Ethical approval was obtained for all samples (Sunderland Research Ethics committee).

### Cell isolation

SF mononuclear cells were obtained by treating samples with 10 U/mL hyaluronidase (Hyalase) and 1 U/mL heparin for 30 min before performing density centrifugation (Lymphoprep, Greiner BioOne). Synovial tissue samples were cut into small fragments and digested overnight in 256 U/mL collagenase type-IV (Worthington) before passing through a 70 μm filter. Macrophage/DC subsets (CD1c+ and CD16+) were separated by flow-assisted cell-sorting (FACS Fusion, Becton-Dickinson). Whole CD4+ T cells were isolated by magnetic bead cell sorting (CD4+ Microbeads, Miltenyi Biotec) with >95% purity. Naïve CD4+ T cells were isolated by magnetic bead cell sorting using the EasySep Human Naïve CD4+ T-cell enrichment kit (StemCell) with >95% purity.

### Cell surface markers

Single cell suspensions were incubated in a buffer solution of phosphate-buffered saline+3% fetal calf serum (Lonza)+0.2% EDTA+0.1% sodium azide. Cells were incubated with antibodies and 4% human IgG for surface staining.

### Cytokine production

Synovial mononuclear cellular fractions at a concentration of 10^6^/mL were stimulated with 0.1 μg/mL lipopolysaccharide (LPS) or 10 ng/mL phorbol 12-myristate 13-acetate (PMA) and 1 μg/mL ionomycin (all from Sigma). Synovial fibroblasts were cultured to confluency (5×10^4^/mL), treated for 3 h with IL-1β (10 ng/mL, Peprotech), washed and cultured for another 24 h. GM-CSF levels in supernatants were determined by BD Biosciences OptEIA ELISA according to the manufacturer's instructions. For intracellular cytokine staining, CD4+ T cells were stimulated with PMA and ionomycin for 5 h with 10 μg/mL Brefeldin A (Sigma) added after 1 h. Cells were then harvested, fixed and permeabilised and stained using the FoxP3/transcription factor staining buffer set (eBioscience) according to the manufacturer's instructions. Cells were preincubated with 2% mouse and rat serum (both Sigma) prior to antibody labelling.

### Flow cytometry

#### T helper cell polarisation

Naïve or unfractionated CD4+ T cells were cultured in a 96-well plate at 5×10^5^/mL in Iscove's modified Dulbecco's medium supplemented with 10% Serum Replacement (both Life Technologies; see online supplementary data). Cells were stimulated with anti-CD2/CD3/CD28-coated beads (Miltenyi Biotec; prepared according to the manufacturer's instructions) at 1 bead:10 cells ratio. All cytokines were added at a concentration of 20 ng/mL on days 0 and 3. Cytokines were purchased from Immunotools (IL-12 and IL-13), Cambridge Bioscience (IL-15, IL-18) and Peprotech (IL-1β, IL-6, transforming growth factor-β (TGFβ)). Human recombinant tumor necrosis factor (TNF)-α was kindly provided by Knoll AG (Ludwigshafen, Germany). Cells were inspected daily and split as required. On day 6, cells were rested in IL-2 (10 U/mL) for a further 4 days before being prepared for intracellular cytokine labelling as described above.

#### Morphological analysis

Cytospin slides were made of sorted cellular fractions.

#### Statistical analysis

All statistical analyses were performed with GraphPad Prism software. Analysis of the difference between two groups was assessed by paired t test, while comparisons between three or more groups were assessed using a one-way repeated measures analysis of variance with post-hoc analysis using Tukey's multiple comparison test.

## Results

### CD4+ T cells are a major source of GM-CSF in RA and are primed to produce it

**Figure 1:**
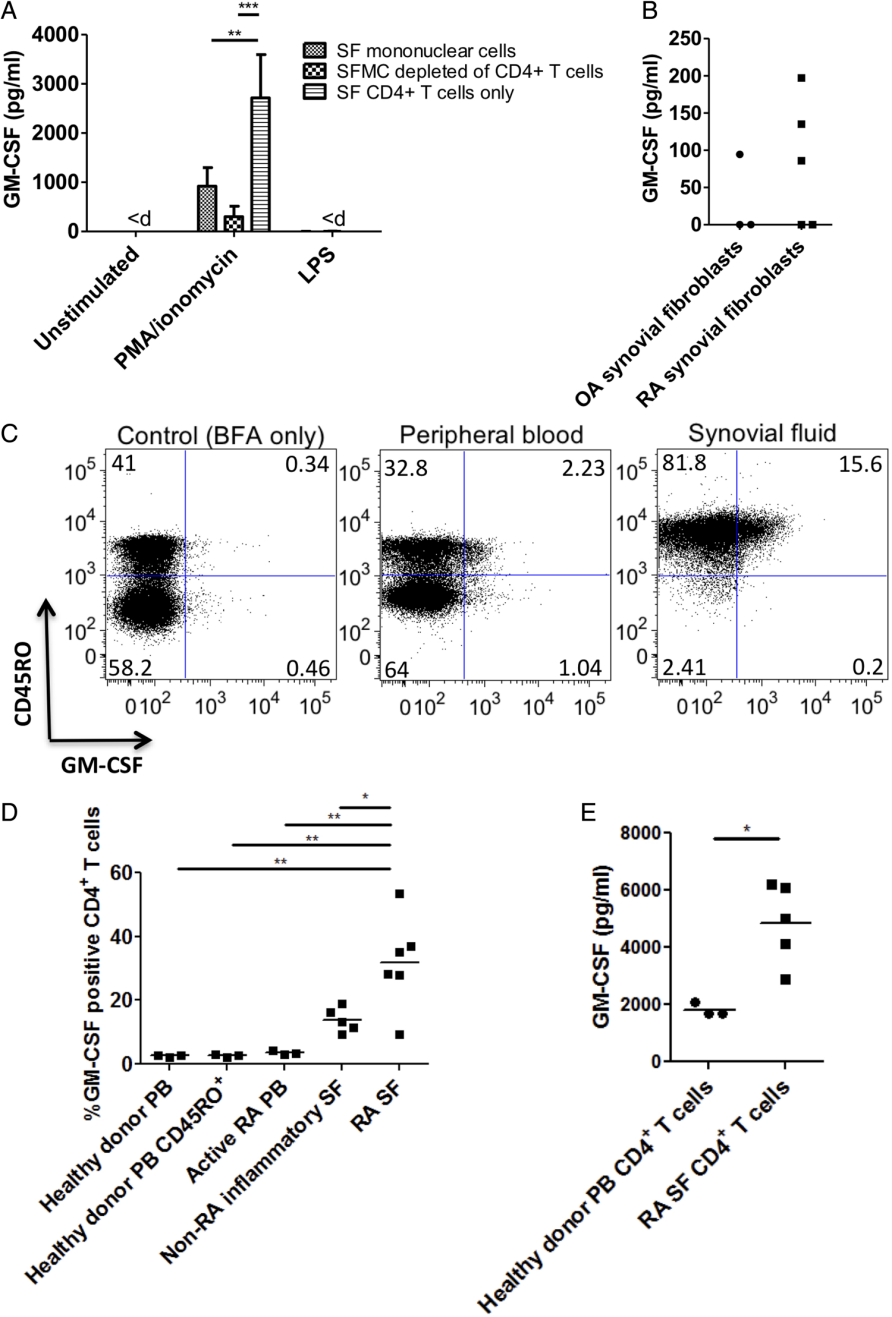
CD4+ T cells are the main source of granulocyte macrophage-colony-stimulating factor (GM-CSF) in rheumatoid arthritis (RA) and are primed to produce it. (A) RA synovial fluid (SF) mononuclear cell (MC) fractions (whole SFMC, SF CD4+ T cells and SFMC depleted of CD4+ T cells; 10^6^ cells/mL) were left unstimulated or were stimulated with phorbol 12-myristate 13-acetate (PMA; 10 ng/mL) and ionomycin (1 μg/mL) or lipopolysaccharide (LPS; 100 ng/mL) for 12 h. GM-CSF levels in supernatants were determined by ELISA. Data are represented as mean±SEM of six independent donors. Results below the limit of detection of the ELISA (4.7 pg/mL) are indicated by ‘<d’. Data were analysed by a two-way ANOVA followed by Bonferroni post-test analysis. (B) GM-CSF levels in the supernatant of confluent synovial fibroblast cultures (5×10^4^ cells/mL) stimulated with interleukin-1β for 24 h were measured by ELISA. (C and D) Peripheral blood (PB) and SF CD4+ T cells (10^6^ cells/mL) were stimulated with PMA/ionomycin in the presence of brefeldin A for 5 h before surface staining with anti-CD45RO antibody and analysis of GM-CSF expression by intracellular cytokine staining followed by flow cytometry. Representative flow data are shown in (C) and data of four to five donors per group are presented in (D). Data were analysed by one-way ANOVA with post hoc analysis by Tukey's multiple comparison testing. Horizontal bars represent mean values. (E) PB and RA-SF CD4+ T cells (10^6^ cells/mL) were stimulated with PMA/ionomycin overnight and GM-CSF levels in supernatants were analysed by ELISA. Horizontal bars represent mean values. Statistical analysis was performed by a two-tailed t test. *p<0.05, **p<0.01 or ***p<0.001.

To define cellular source(s) of GM-CSF in RA-SF, we separated RA-SF cells into three fractions: whole SF mononuclear cells, SF CD4+ T cells and SF mononuclear cells depleted of CD4+ T cells. Each fraction was stimulated with either LPS or PMA/ionomycin to ensure activation of the myeloid and lymphoid compartment (figure 1A). LPS stimulation did not result in detectable GM-CSF production from any fraction. We also found that CD4+ T cells alone were able to produce significantly more GM-CSF than CD4+ depleted SF mononuclear cells and whole SF mononuclear cells.

Synovial fibroblasts have been identified as a significant source of GM-CSF.^31^ We assessed their contribution by stimulating cultured synovial fibroblasts with IL-1β and TNFα, two cytokines produced by synovial macrophages and thought to be responsible for the activated phenotype of RA synovial fibroblasts.^32^ Whereas levels of GM-CSF were undetectable following stimulation with up to 50 ng/mL TNFα (data not shown), IL-1β was an effective stimulus for GM-CSF production (figure 1B). Some but not all OA and RA synovial fibroblasts produced detectable levels of GM-CSF, although this was consistently <250 ng/mL.

We next examined whether RA synovial CD4+ T cells are ‘primed’ to produce GM-CSF or whether this is a property of CD4+ T cells in general by comparing them with PB CD4+ T cells. As with other cytokines (with the exception of IL-2),^33^ the majority of GM-CSF+ T cells in PB were CD45RO+ memory T cells (figure 1C). We compared the proportion of GM-CSF+ CD4+ T cells in the PB of healthy donors, patients with active RA (defined as DAS28 >5.1), non-RA inflammatory SF and RA-SF (figure 1D) and found that RA-SF contained a significantly higher proportion of GM-CSF+ T cells. This was verified by analysing supernatants of stimulated PB and RA-SF CD4+ T cells by ELISA (figure 1E; right panel).

### Human CD4+ T cells optimally produce GM-CSF under high stimulus, Th1 conditions

**Figure 2:**
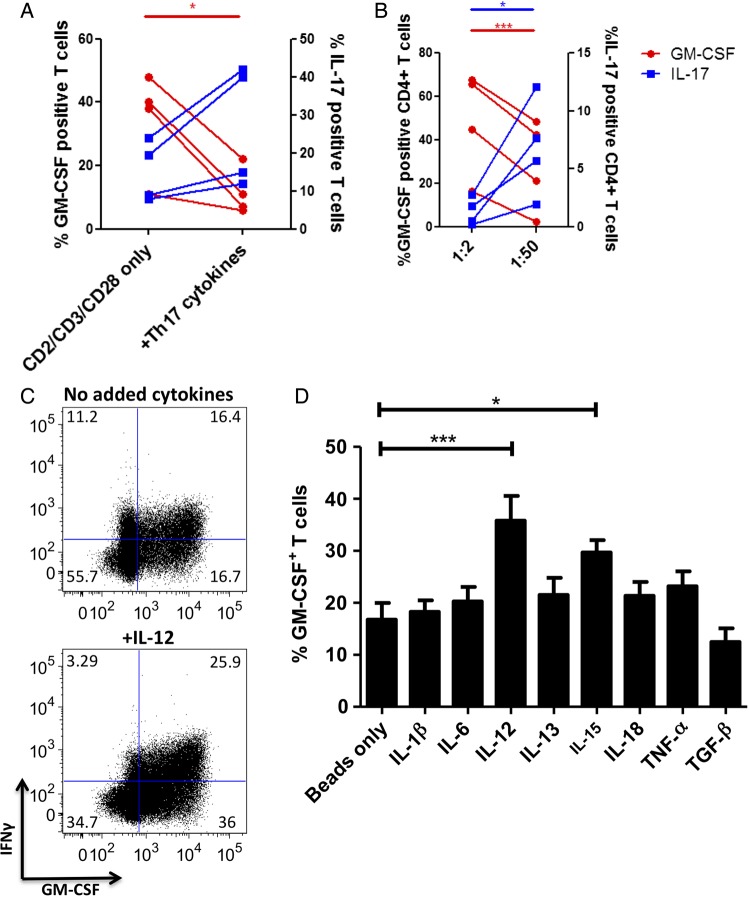
Human CD4+ T cells optimally produce granulocyte macrophage-colony-stimulating factor (GM-CSF) under high stimulus Th1 conditions. (A) Peripheral blood (PB) CD4+ T cells (5×10^5^ cells/mL) from healthy donors were stimulated with anti-CD2/CD3/CD28-coated beads without or with Th17 polarising cytokines (interleukin (IL)-1β, transforming growth factor-β (TGF-β) and IL-23, all at 20 ng/mL) at a ratio of 1:10 beads:T cells for six days before being rested in IL-2 (10 U/mL) for a further 4 days. Cytokine production was analysed by flow cytometry. Results of four independent experiments are shown. Statistical analysis was performed by a paired two-tailed t test. (B) PB CD4+ T cells (5×10^5^ cells/mL) from healthy donors were stimulated with anti-CD2/CD3/CD28-coated beads at indicated ratios of cells to beads for six days before being rested in IL-2 (10 U/mL) for a further 4 days. Cytokine production was analysed by flow cytometry. Results of four independent experiments are shown. Statistical analysis was performed by a paired two-tailed t test. (C and D) Naïve healthy donor PB CD4+ T cells (5×10^5^ cells/mL) were stimulated with anti-CD2/CD3/CD28-coated beads in the presence of the indicated cytokines (all at a concentration of 20 ng/mL, added day 0 and refreshed on day 3) and on day 6 the cells were rested in IL-2 (10 U/mL) for a further 4 days before analysis of cytokine production by intracellular flow cytometry. Representative flow data are shown in (C) and data of seven independent donors per group are presented in (D) with the results analysed by one-way followed by post hoc analysis by Tukey's multiple comparison testing. *p<0.05, **p<0.01 or ***p<0.001.

We next sought to identify the cytokines present in the RA synovium that may enhance GM-CSF production by T cells by performing in vitro polarisation experiments. In the context of the description of GM-CSF as a ‘Th17 cytokine’ in mice,^28^ we first assessed the effect of Th17-promoting cytokines (IL-1β, TGFβ and IL-23) on GM-CSF induction in total CD4+ T cells. IL-23 alone did not affect the proportion of GM-CSF+ T cells (data not shown) and in combination Th17-promoting cytokines resulted in a lower proportion of GM-CSF+ T cells (figure 2A). We next investigated how T-cell stimulation strength modulates GM-CSF production. We have previously demonstrated that low stimulation strength supports Th17 responses^34^ and as expected found that IL-17 levels increased as stimulatory CD2/CD3/CD28 bead concentration decreased. In contrast, GM-CSF production increased at higher bead concentrations (figure 2B). We next sought to identify the cytokines that support GM-CSF production with reference to those found in RA using naïve CD4+ T cells and the same experimental set up. We found that GM-CSF production was significantly enhanced by IL-12 and IL-15 (figure 2C, D), but not by Th17-promoting cytokines (IL-1β, IL-6, TGFβ), IL-13 and TNFα. These findings suggest that the factors that enhance human GM-CSF production are different from those for mice. Specifically, GM-CSF production in human CD4+ T cells is enhanced by a strong T-cell activation stimulus, Th1 conditions and IL-15.

### RA synovial CD4+ T cells promote CD14+ monocytes to differentiate into a CD1c+ population and this effect is GM-CSF dependent

**Figure 3:**
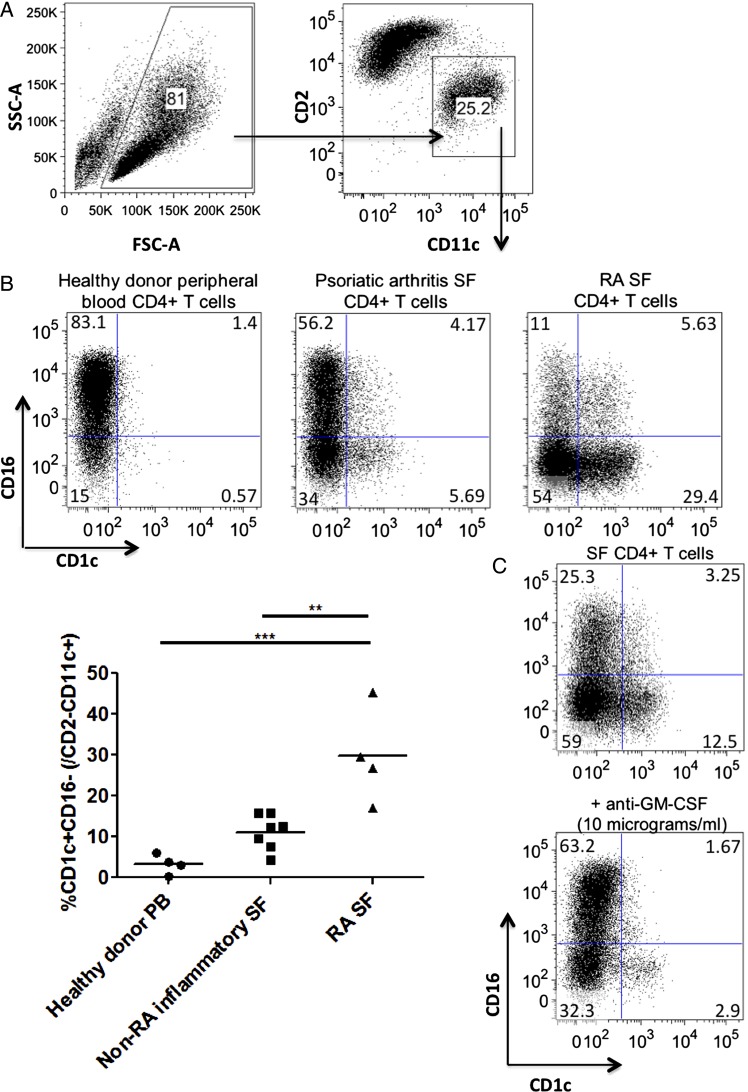
Rheumatoid arthritis (RA) synovial CD4+ T cells promote CD14+ monocytes to differentiate into a CD1c+ population and this effect in granulocyte macrophage-colony-stimulating factor (GM-CSF) dependent. (A) CD4+ T cells were isolated from healthy donor peripheral blood (PB), non-RA inflammatory arthritis and RA synovial fluid (SF) and cultured together with allogeneic healthy donor CD14+ monocytes in a 24-well plate at a ratio of 1:2 monocytes:T cells (5×10^5^ monocytes:10^6^ T cells/mL) for 3 days. Cells were harvested and cell surface marker expression was analysed by flow cytometry using the gating strategy indicated (top) with representative examples of flow data (bottom). The proportion of CD16−CD1c+ cells within the CD2−CD11c+ fraction induced by different CD4+ T-cell donors was analysed. Results from four to seven donors per group are summarised in (B) with horizontal bars representing mean values. Data were analysed by ANOVA before post hoc analysis by Tukey's multiple comparison testing. (C) SF T cells were cocultured with allogeneic monocytes as above in the presence of a neutralising antibody to GM-CSF at 10 μg/mL. Representative results from two independent repeats are shown. *p<0.05, **p<0.01 or ***p<0.001.

We next sought to assess the functional significance of this enhanced GM-CSF production by synovial CD4+ T cells. We cultured CD4+ T cells isolated from PB, non-RA inflammatory SF and RA-SF with healthy donor allogeneic CD14+ monocytes to model mutual activation of these cells when monocytes infiltrate the joint. After 3 days we determined the fate of the monocytes within the myeloid CD2^lo^CD11c+ fraction (figure 3A). We found that, in contrast to healthy PB CD4+ T cells, psoriatic arthritis and in particular RA-SF CD4+ T cells supported the development of a population of CD1c+ DCs. CD1c is a lipid antigen presentation molecule used to define a subset of myeloid DC and more recently shown to be expressed by infDCs in RA-SF.^21^ RA CD4+ T cells promoted significantly higher levels of CD1c+ differentiation than non-RA inflammatory arthritis CD4+ T cells (figure 3B). To assess whether this effect was GM-CSF dependent, we neutralised GM-CSF using a blocking antibody and found a significant reduction in the CD1c+ population (figure 3C).

### The CD1c+ cells induced by RA-SF T cells have phenotypic and functional properties of DCs

**Figure 4:**
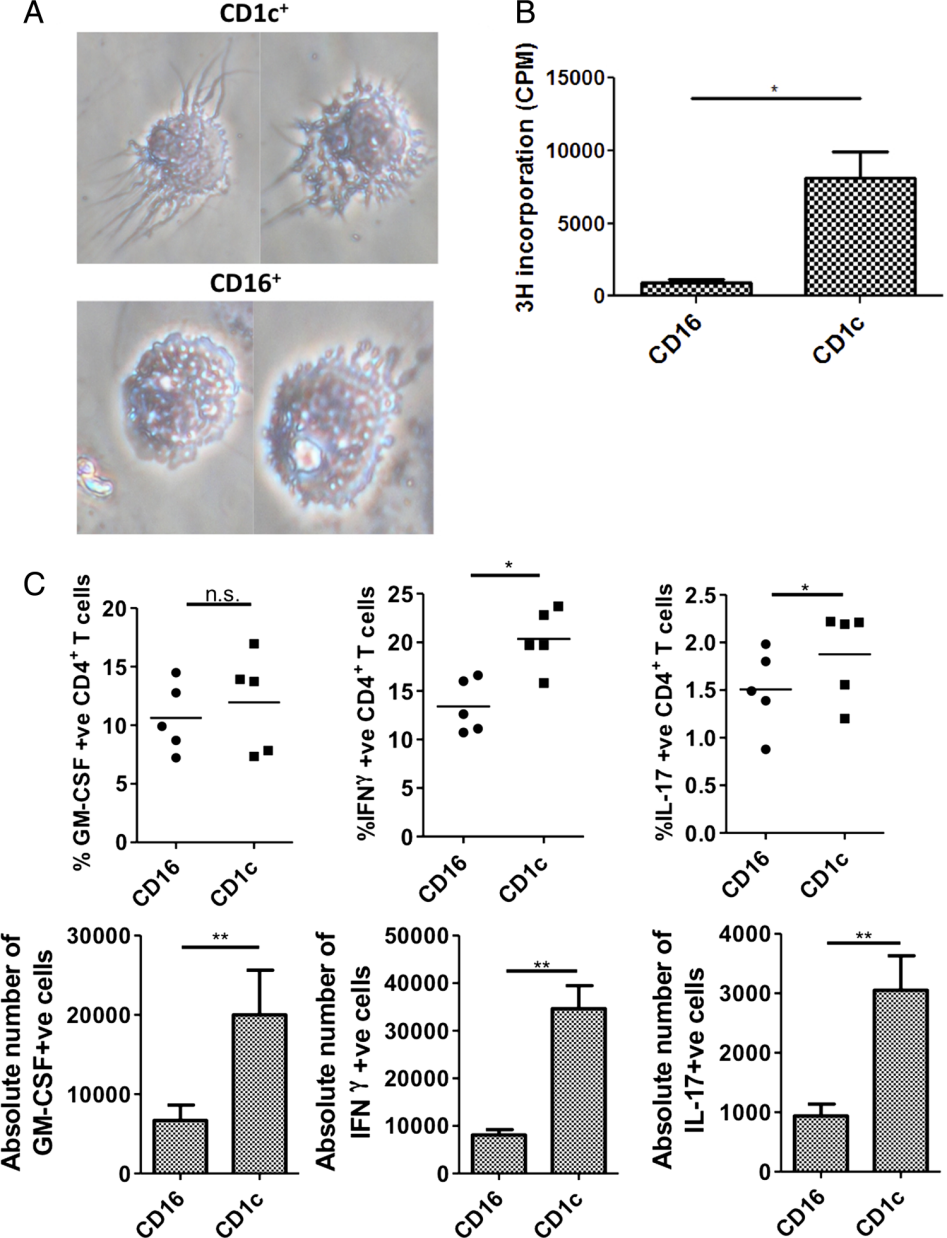
The CD1c+ population induced by rheumatoid arthritis synovial fluid (SF) T cells have phenotypic and functional properties of dendritic cells. (A) CD16+CD1c− (‘CD16’) and CD16−CD1c+ (‘CD1c’) fractions were flow-sorted from 3-day cocultures of CD14+ monocytes and allogeneic SF CD4+ T cells and phenotype assessed by phase-contrast microscopy of cytospin slides. (B) The same flow-sorted fractions were placed in an allogeneic mixed lymphocyte reaction (MLR; 10^4^ antigen presenting cells/well) in a 96-well plate with CD4+ T cells at a 1:10 ratio for 6 days. T-cell proliferation was analysed at this point by ^3^H-thymidine incorporation assay. Results are shown as mean±SEM of four independent donors (CPM = counts per minute). Data were analysed by a two-tailed t test. (C) CD4+ T cells from the same MLRs were harvested on day 6, counted to provide an absolute number and cytokine production analysed following restimulation with phorbol 12-myristate 13-acetate/ionomycin (10^6^ cells/mL) by intracellular cytokine staining and flow cytometry. The percentage cytokine positive cells (top, n=5, top, horizontal lines represent mean values) and the absolute number of cytokine producing cells (bottom, n=4, results shown as mean±SEM) are shown. Analysis was performed by paired two-tailed t test. *p<0.05, **p<0.01 or ***p<0.001. IFN, interferon; IL, interleukin.

Having demonstrated that RA synovial CD4+ T cells promoted differentiation of monocytes into a CD1c+ population, we next sought to assess whether this induced population had DC characteristics. Using the same gating strategy as above, we performed flow sorting on the CD1c−CD16+ (hereafter ‘CD16+’) and CD1c+CD16− (hereafter ‘CD1c+’) cells derived from those culture conditions. The CD16+ population had ruffled edges and prominent vacuoles consistent with macrophage morphology, whereas the CD1c+ population developed fine dendritic processes consistent with DC morphology (figure 4A). We compared the expression of surface markers that have previously been identified on RA CD1c+ DCs.^21^ ^29^ We found that the expression of CD14, CD11b, CD206 and Signal-regulatory protein alpha (SIRPα) was concordant between RA-SF and synovial tissue CD1c+ DCs and our induced CD1c+ population (see online supplementary figure S1) but expression of both FcεRI and Dendritic Cell-Specific Intercellular adhesion molecule-3-Grabbing Non-integrin (DC-SIGN) was lower. This suggests that our in vitro model cannot fully recapitulate moDC generation in vivo. In an allogeneic mixed lymphocyte reaction (MLR) with CD4+ T cells the CD1c+ population induced significantly greater proliferation than the CD16+ population (figure 4B). Finally, we assessed the capacity of the two populations to promote CD4+ T-cell polarisation. Both promoted cytokine production by T cells and significantly higher proportions of IFNγ+ and IL-17+ cells were induced in MLRs with the CD1c+ population (figure 4C, top). As expanded T-cell numbers were higher in the CD1c+ cultures the absolute numbers of IFNγ+, IL-17+ and GM-CSF+ cells were also higher (figure 4C bottom). These data support the idea that the induced CD1c+ population possesses infDC characteristics.

### RA-SF is enriched for a population of CD1c+ cells within the macrophage/DC fraction

**Figure 5:**
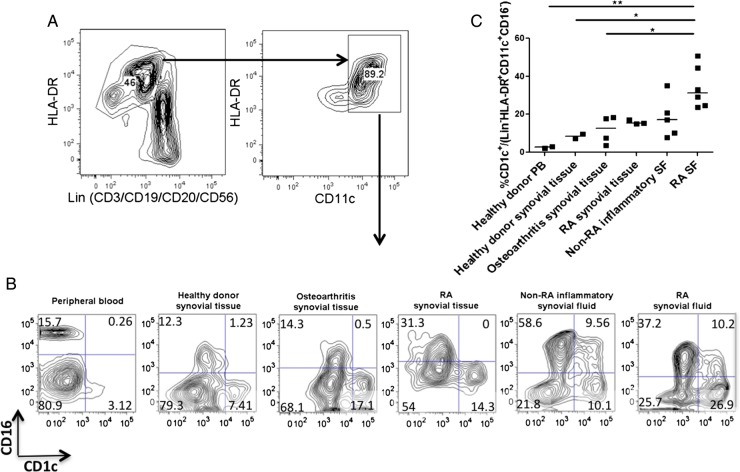
Rheumatoid arthritis (RA) synovial fluid (SF) is enriched for a population of CD1c+ cells within the macrophage/dendritic cell fraction. (A–C) Single cell suspensions were obtained from digested synovial tissue and compared with SF and blood using the strategy indicated in (A) to compare the proportions of CD16+CD1c− and CD16−CD1c+ cells within the lin^−^HLA-DR+CD11c+ fraction. Representative examples shown in (B) and a summary of two to six donors for each condition summarised in (C). Horizontal lines represent mean values. Groups were analysed by one-way ANOVA and post-hoc analysis was performed using Tukey's multiple comparison test. *p<0.05, **p<0.01 or ***p<0.001.

We have demonstrated that RA-SF CD4+ T cells induce the differentiation of a population of CD1c+ cells with infDC characteristics in a GM-CSF-dependent manner. Finally, we sought to correlate this with the situation in vivo: specifically whether a higher proportion of CD1c+ cells were found in the macrophage/DC fraction of SF cells in RA than in tissue from healthy controls and non-RA inflammatory arthritis. We used a lineage cocktail with CD3, CD19, CD20 and CD56 to exclude T cells, B cells and natural killer cells and hence identified the macrophage/DC fraction as DAPI^−^CD45+lin^−^HLA-DR+CD11c+ (gating figure 5A; representative data figure 5B; summarised figure 5C). In agreement with the previous work of Moret *et al*^6^ and consistent with our in vitro finding that RA-SF CD4+ T cells promote differentiation of monocytes into infDC-expressing CD1c, we found that a significantly higher proportion of myeloid cells in RA-SF was CD1c+ DC than in the other conditions examined. However, we did not find a significant enrichment of CD1c+ DC in RA synovial tissue compared with healthy and OA tissue. Lebre *et al*^29^ have previously demonstrated no significant difference in CD1c+ DC in RA and inflammatory OA. A possible explanation for our finding could be that tissue DC migrates into SF in inflammation.

## Discussion

We demonstrate that synovial CD4+ T cells are a major source of GM-CSF in RA. We show that GM-CSF production by human CD4+ T cells is enhanced by the Th1-polarising cytokine IL-12 and the T-cell survival factor IL-15 and confirms the recent findings by Noster *et al*^35^ This correlates with the cytokine environment CD4+ T cells are likely to encounter in vivo and may explain our finding that ex vivo RA synovial T cells produce more GM-CSF than RA or healthy control PB CD4+ T cells. Levels of IL-12 have been shown to be increased in serum and SF of patients with RA compared with osteoarthritis and correlate with disease activity score.^36^ IL-15 can be found in RA-SF but not in OA SF^37^ and levels also correlate with disease activity.^38^ Both IL-12 and IL-15 have been shown to be produced by synovial CD1c+ DC by immunohistochemistry.^29^ Lymphocytes are closely associated with CD1c+ cells in RA synovial tissue^29^ and this suggests a mechanism by which production of IL-12 and IL-15 by these cells contributes to disease through the development of a positive feedback loop characteristic of chronic inflammatory state such as RA.

These findings suggest that the regulation of GM-CSF production by CD4+ T cells differs between mice (where it has been shown to be regulated by RORγt and as such designated a Th17 cytokine^28^) and humans. Our finding that IL-12 enhances GM-CSF production accords with a recent study showing that GM-CSF production increases under Th1 conditions and that transcription of GM-CSF and IL-17 is reciprocally regulated with a high STAT5:STAT3 ratio supporting GM-CSF production and suppressing IL-17 and vice versa.^35^ In juvenile idiopathic arthritis, another inflammatory arthritis, the majority of GM-CSF-producing synovial CD4+ T cells express CD161, a marker for ex-Th17 cells.^39^ ^40^ It has been shown that human Th17 cells develop combined IL-17/IFNγ/GM-CSF-producing capacity under the influence of IL-12 and this may be responsible for their pathogenicity.

Based on our findings, we propose that human CD4+ T-cell-derived GM-CSF can support differentiation of monocytes in to infDC-expressing CD1c. However, while GM-CSF is required in this model, other factors are also likely to contribute. It has previously been shown that T helper cells support monocyte to DC differentiation through cell-to-cell contact and production of TNFα as well as GM-CSF.^41^ In mice there has been conflicting evidence for the role of GM-CSF in murine infDC differentiation. Greter *et al*^22^ demonstrated that in csf2r^−/−^ mice, infDC (defined as MHC-II+CD11c^int^CD11b+Ly6c+) developed normally in response to influenza and *Streptococcus pneumoniae* while Campbell *et al*^42^ have shown that infDCs defined by the same surface markers are absent from the synovial tissue and lymph nodes of GM-CSF^−/−^ mice after induction of acute monoarticular arthritis. These data suggest that the requirement for GM-CSF for infDC differentiation differs between murine models and other factors may be substituted. For example, IFNγ is required for infDC differentiation in the context of *Toxoplasma gondii* infection.^43^

We find an enriched CD1c+ population in RA-SF but we cannot conclude that they are monocyte-derived infDC as they cannot be distinguished from steady-state DC by surface marker analysis alone. Despite this there is evidence that infDC will comprise the majority of this population. In murine acute inflammatory arthritis, 85% of the CD11c+ population in synovial tissue have been previously shown to be infDC.^42^ In humans, the gene signature of RA-SF CD1c+ DCs is closest to that of moDC, suggesting that infDCs predominate.^21^

The specific contribution of human infDCs to RA pathogenesis is uncertain. Murine infDCs are effective at inducing T-cell proliferation and producing inflammatory cytokines such as IL-12, IL-23 and TNFα^17^ ^19^ ^44^ but poor at migrating to draining lymph nodes.^19^ ^45^ Similarly, in our study, synovial CD4+ T-cell-induced infDCs display potent T-cell stimulatory ability and enhance cytokine production, but it is not clear whether they have the capacity to migrate to draining lymph nodes. Analogous to murine infDC the role of human infDC in RA may be to perpetuate T-cell responses within the synovium, a finding supported by the demonstration of mature DC within lymphocytic infiltrates in synovial tissue.^46^

In summary, we have demonstrated a mechanism by which RA synovial CD4+ T cells can support infDC differentiation through production of GM-CSF. This provides both a novel indication of how GM-CSF may contribute to the maintenance of synovial inflammation and a model for examining RA infDC development. The development of biological agents targeting GM-CSF in RA should allow us to validate these findings in vivo.

## Supplementary Material

Web figure
