# Supplementary material for: Synovial CD4+ T-cell-derived GM-CSF supports the differentiation of an inflammatory dendritic cell population in rheumatoid arthritis
Source: Ann Rheum Dis. 2015 Apr 28;75(5):899–907. doi: 10.1136/annrheumdis-2014-206578 (PMC4853576; doi:10.1136/annrheumdis-2014-206578)
Supplement: Web figure [file annrheumdis-2014-206578-s1.pdf]

Supplementary figure 1 Expression of indicated cell surface markers was compared between CD1c+ (blue line) and CD16+ (green line) populations as per the gating in figure 5 in peripheral blood, RA synovial fluid, RA synovial tissue and monocytes following culture with RA CD4+ T cells.

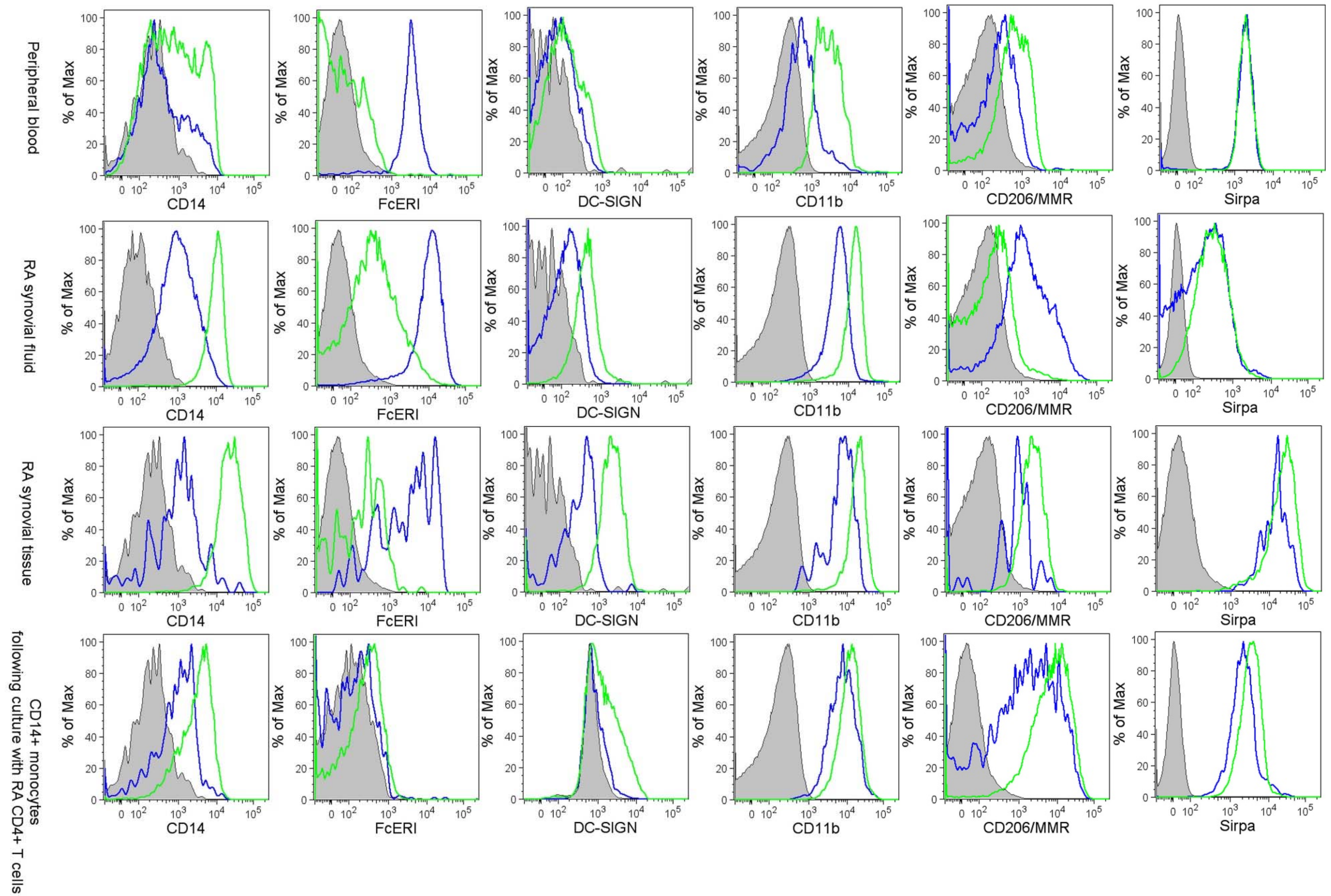
Peripheral blood
RA synovial fluid
RA synovial tissue
CD14+ monocytes following culture with RA CD4+ T cells
% of Max
CD14
FcERI
DC-SIGN
CD11b
CD206/MMR
Sirpa
